# Evidence for a Neural Stem Cell Niche in the Postnatal Rat Vestibular Ganglion: An In Vitro Study

**DOI:** 10.1155/sci/5567583

**Published:** 2026-09-06

**Authors:** Sabine Sommerer, Kristen Rak, Bjoern Spahn, Stephan Hackenberg, Johannes Voelker, Jonas Engert

**Affiliations:** ^1^ Department of Otorhinolaryngology, Head and Neck Surgery, University Hospital Wuerzburg, Wuerzburg, Germany, uk-wuerzburg.de

**Keywords:** endogenous neural stem cells, neurosphere, regenerative vestibular therapy, Scarpa’s ganglion, vestibular system

## Abstract

Vestibular dysfunction significantly contributes to dizziness, balance issues, and falls, yet causal treatments are still unavailable. The vestibular ganglion (VG), the primary sensory ganglion of the vestibular nerve, is increasingly seen as a key site of vestibular degeneration. Neural stem cells (NSCs) have been identified in several areas of the auditory and vestibular pathways. Endogenous activation of NSCs represents a promising regenerative strategy. However, the presence of NSCs within the VG remains uncertain. In this study, VG tissue from postnatal day (PND) 8 Sprague‐Dawley rats was isolated, enzymatically dissociated, and cultured in serum‐free neurosphere conditions. The dissociated VG cells reliably formed free‐floating neurospheres that grew over multiple passages. Immunocytochemistry showed expression of NSC and progenitor markers, including Sox‐2, Nestin, Musashi‐1, Atoh‐1, and doublecortin, in both neurospheres and VG tissue sections. Additional analyses demonstrated persistence of Sox‐2‐, Atoh‐1‐, and Nestin‐positive populations in the adult VG. After differentiation, individual cells from neurospheres developed into β‐III‐tubulin‐positive neurons, glial fibrillary acidic protein (GFAP)‐positive astrocytes, and myelin basic protein (MBP)‐positive myelinating glial cells, while a significant portion retained Nestin expression, indicating a progenitor‐like state. These findings demonstrate that VG‐derived cells meet the main criteria of NSCs: self‐renewal, progenitor formation, and multipotent differentiation into neuronal and glial lineages. Our results provide the first evidence of a neurogenically capable cell population in the postnatal rat VG and support the persistence of progenitor‐associated cell populations into adulthood. This identifies the VG as a novel endogenous regenerative reservoir, positioning it as a promising target for strategies that activate resident cells to restore vestibular function.

## 1. Introduction

Vestibular dysfunction is a common, clinically significant, and financially impactful condition. In a large US population‐based survey, 35.4% of adults aged 40 years or older showed objective signs of vestibular impairment, and its prevalence increased notably with age [1]. Fall‐related injuries among older adults, for which vestibular dysfunction is a key risk factor, create a substantial economic burden. In the United States, direct medical costs for nonfatal falls among individuals aged 65 or older reached ~19 billion dollars in a single year [2].

The histopathological basis of age‐related decline of the vestibular system remains debated as multiple vestibular structures may be affected [3]. Temporal bone studies report significant age‐related loss of type I and type II hair cells, particularly in the semicircular canal cristae, whereas other analyses show only modest or region‐specific changes, including largely preserved utricular hair cell numbers [4–8]. In contrast, degeneration of the human vestibular ganglion (VG) appears more consistent with aging. The VG contains the bipolar first‐order neurons of the vestibular nerve [9, 10]. Human temporal bone studies demonstrate a progressive decline of VG neurons, with an estimated loss of ~57 cells per year and a total reduction of about 20%–25% in older adults [11, 12]. Together, these findings identify VG degeneration as a key structural contributor to age‐related vestibular dysfunction.

Although age‐related degeneration is a major contributor, vestibular dysfunction also arises from congenital defects, genetic disorders, or early life inner ear pathology. As a recent meta‐analysis reported a global prevalence of ~30% of vestibular dysfunction in children and adolescents [13]. Neurodegenerative syndromes such as cerebellar ataxia with neuropathy and bilateral vestibular areflexia (CANVAS) demonstrate that vestibular dysfunction results from neuropathy of the sensory ganglia and nerve [14]. Children with congenital hearing loss also present vestibular nerve deficiency together with measurable vestibular dysfunction [15]. Moreover, in pediatric and young adult patients with vestibular schwannomas, including those associated with neurofibromatosis type 2, surgical management frequently entails the sacrifice of the vestibular nerve to achieve durable tumor control, which leads to vestibular dysfunction [16, 17].

Despite the high clinical importance, the available treatment options remain rather limited. In a recent worldwide survey of vestibular specialists, 77% of respondents reported that vestibular physical therapy was their mainstay treatment for patients with bilateral vestibulopathy (BVP) [18]. Vestibular implants are currently being applied in patients with BVP, where artificial stimulation of the vestibular nerve can partially restore vestibulo‐ocular reflex function and improve dizziness‐related symptoms and quality of life. Still, this approach remains experimental and is not established in clinical practice. Thus, the lack of any causal therapy underscores the urgent need for new therapeutic strategies targeting the underlying vestibular dysfunction.

Neural stem cells (NSCs) represent a promising candidate for a regenerative therapeutic approach because they possess the ability to self‐renew and generate new neurons and glial cells in the adult mammalian brain [19]. Neurogenesis refers to the generation of new neurons arising from NSC and progenitor cell populations [20]. NSCs are defined by their capacity for sustained self‐renewal and their multipotency, enabling them to produce neural progenitor cells as well as neurons, astrocytes, and oligodendrocytes [21]. Recent reviews further emphasize that NSCs, whether recruited endogenously or introduced therapeutically, may hold the potential to restore neural circuitry and replace lost cells in neurodegenerative disease [22]. Evidence from models of spinal cord injury shows that endogenous NSC niches can be activated in vivo through modulation of niche signals, trophic support, or environmental stimulation, leading to proliferation, migration, and differentiation of NSCs into functional neural cell types [23]. These findings support a translational strategy that focuses on the activation of resident NSC niches [24].

Adult NSC niches were first identified in the dentate gyrus of the hippocampus and the subventricular zone of the lateral ventricles [25, 26]. Beyond these well‐characterized neurogenic niches, growing evidence suggests that NSCs and progenitor‐like cells also persist in the mature inner ear. It has been demonstrated that pluripotent cells can be isolated from the adult mouse inner ear and, in vitro, give rise to hair‐cell–like and other inner ear cell types [27]. Furthermore, in the human auditory system, adult neural progenitor cells have been cultured from the spiral ganglion and have been shown to have the potential to differentiate into neurons and glial cells [28]. Moreover, NSC niches have been identified at multiple levels of the auditory pathway, highlighting the presence of neurogenic cell populations throughout the auditory system [29–31]. Similar evidence of NSC niches has also been reported in the closely related and anatomically adjacent vestibular system, including vestibular nuclei and vestibular sensory epithelia [32, 33]. Interestingly, neuronal plasticity within the VG has already been demonstrated, supporting the notion that this structure may retain a capacity for structural remodeling and possibly regeneration in adulthood [34]. Additionally, the VG has been surgically accessed and even resected in patients undergoing the removal of vestibular schwannoma, showing that the VG is anatomically reachable by established surgical approaches [35].

Given the relevant role of the VG in vestibular dysfunction, and the lack of any causal therapy, there is a clear need for innovative regenerative treatment strategies. NSCs are a promising approach in this context, especially since the VG has already shown signs of neuronal plasticity and is surgically accessible. Demonstrating a resident NSC niche within the VG could therefore establish a realistic target for future endogenous activation approaches to restore vestibular function. Therefore, this study examines whether VG‐derived cells of the rat display the main criteria of NSCs, providing a foundation for future regenerative ideas.

## 2. Materials and Methods

### 2.1. Animal Preparation

**Figure 1:**
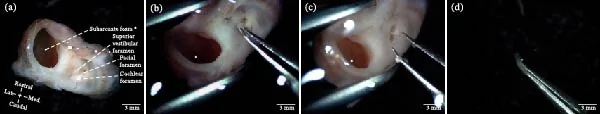
Preparation of VG of a PND 8 rat. (a) Medial view of the left temporal bone after removal of the brain, cerebellum, and brainstem; the subarcuate fossa ( ^∗^) serves as a key anatomical landmark. (b) Identification and blunt dissection of the superior portion of VG within the internal acoustic meatus. (c) View of the internal acoustic meatus following ganglion removal. (d) Isolated VG positioned on the tips of microsurgical forceps.

Postnatal day (PND) 8 and adult Sprague‐Dawley rats (Charles River, Wilmington, MA, U.S.A.) were euthanized by cervical dislocation and decapitation. The skull was opened midsagittally, and the cranial bones were removed. All cranial nerves were transected close to the brainstem to expose the skull base. The brain, cerebellum, and brainstem were completely removed and discarded. The temporal bone was then identified and gently released from the skull base by blunt fingertip pressure, yielding an intact bone fragment. The isolated skull base containing the temporal bone was transferred into a 35 mm Petri dish (CELLSTAR, Greiner Bio‐One, Monroe, NC, U.S.A.) filled with cold Neurobasal medium (Thermo Fisher Scientific, Grand Island, NY, U.S.A.) (Figure 1a). Meningeal tissue and residual vessels were removed from the temporal bone under a stereomicroscope (Stemi 508, ZEISS, Oberkochen, Germany). The dissection was guided by the anatomical orientation described by Curthoys [9]. The subarcuate fossa served as a landmark to locate the superior and inferior VG within the internal acoustic meatus. After slight medial retraction, the superior vestibular nerve and the ridge‐like elevation of the pars superior of VG were visualized (Figure 1b). By applying gentle traction caudally along the transverse bony crest separating the vestibular and cochlear nerve, the pars inferior of VG was removed together with the pars superior (Figure 1c). VG was mobilized as an intact unit with microsurgical forceps (#5/45 Dumont, Montignez, Switzerland) (Figure 1d). All procedures were performed under sterile conditions.

All experiments were conducted according to the national guidelines for the care and use of laboratory animals (§8) and carried out exclusively as organ removal. Removing organs from the animal after sacrifice is, as per § 6 Abs. 1 No. 4 (German Animal Welfare Act), subject to a notification requirement but has not been and cannot be approved as an animal experiment.

The number of animals sacrificed per species per year must be provided to the local authorities. Accordingly, nine sacrificed Sprague‐Dawley rats were reported to the “Regierung of Unterfranken” (Government of Lower Franconia).

### 2.2. Neurosphere Assay, Cell Culture, and Passaging

Following tissue preparation, samples were enzymatically dissociated in Accutase (Gibco, Thermo Fisher Scientific, Grand Island, NY, U.S.A.) using a ThermoMixer (Eppendorf, Hamburg, Germany) at 37°C and 500 rpm. Every 10 min, gentle trituration was performed until a homogeneous single‐cell suspension was obtained. The suspension was centrifuged at 1000 rpm for 5 min (Centrifuge 5810, Eppendorf, Hamburg, Germany). The resulting pellet was resuspended in Neurobasal medium (Gibco, Thermo Fisher Scientific, Grand Island, NY, U.S.A.) with 1% GlutaMAX (Gibco, Thermo Fisher Scientific, Grand Island, NY, U.S.A.), 2% B27 supplement without retinoic acid (Gibco, Thermo Fisher Scientific, Grand Island, NY, U.S.A.), and 1% penicillin–streptomycin (Gibco, Thermo Fisher Scientific, Grand Island, NY, U.S.A.). Recombinant murine EGF and FGF‐2 (PeproTech, Thermo Fisher Scientific, Grand Island, NY, U.S.A.) were added at a final concentration of 10 ng/mL. This formulation is referred to as the NSC‐medium. Cell viability and total counts were determined using trypan blue (Thermo Fisher Scientific, Grand Island, NE, U.S.A.) in an improved Neubauer hemocytometer (ZK06, Hartenstein, Würzburg, Germany).

Free‐floating neurosphere cultures were maintained in hydrophobic cell culture flasks (CELLSTAR, filter top, 25 cm^2^, Greiner Bio‐One, Monroe, NC, U.S.A.) at 37°C and 5% CO_2_. Cultures were initiated in 4 mL of NSC‐medium, and every 3 days, 2 mL of fresh NSC‐medium was added. At days 4, 8, 12, 16, and 20, representative images of the neurospheres were acquired using an inverted microscope (Leica DMI 4000B/DMI‐8, Wetzlar, Germany) at 20× magnification to assess neurosphere diameter and morphological development.

After 14 days of culture, neurospheres were passaged to investigate proliferation capacity. For passaging, neurospheres were carefully dissociated mechanically using a 10 mL serological pipette (CELLSTAR, Greiner Bio‐One, Monroe, NC, U.S.A.) to obtain single cells, ensuring minimal mechanical stress. The suspension was then centrifuged at 1000 rpm for 5 min and resuspended in fresh NSC‐medium. Cultures were maintained under identical conditions (37°C, 5% CO_2_) for 14 days in 25 cm^2^ filter‐top flasks (CELLSTAR, filter top, 25 cm^2^, Greiner Bio‐One, Monroe, NC, U.S.A.). The number and proportion of viable cells at each passage were determined as described above using trypan blue staining and counting with the improved Neubauer hemocytometer.

### 2.3. Plating of Neurospheres and Single Cells

Neurospheres from free‐floating cell cultures were carefully collected using a 5‐mL automatic pipette (Multipette plus, Eppendorf, Hamburg, Germany) to avoid mechanical disruption. 100 μL of the neurosphere suspension was subsequently plated onto glass coverslips coated with poly‐D‐lysine (100 μg/mL, Serva Electrophoresis, Thermo Fisher Scientific, Grand Island, NE, U.S.A.) and laminin‐1 (10 μg/mL, BD Biosciences, Bergen County, NJ, U.S.A.). The integrity of the neurospheres after transfer was verified under a transmitted light microscope (Leica DMI 4000 B, Wetzlar, Germany). In 4‐well culture dishes (Greiner Bio‐One, Monroe, NC, U.S.A.), 3 mL of NSC‐medium was added per coverslip, and the spheres were incubated at 37°C and 5% CO_2_ for the designated culture period before further analysis.

Single cells were obtained from propagated neurosphere cultures by mechanically dissociating the spheres using a 10 mL serological pipette (CELLSTAR, Greiner Bio‐One, Monroe, NC, U.S.A). The suspension was then centrifuged at 1000 rpm for 5 min, after which the supernatant was carefully removed. The resulting pellet was washed once with DPBS to eliminate the residual culture medium. Following centrifugation, the dissociated cells were resuspended in fresh NSC‐medium. After transfer, the cultures were examined using a transmitted light microscope (Leica DMI 4000 B, Wetzlar, Germany) to ensure complete neurosphere dissociation and the presence of single cells without residual aggregates.

For differentiation assays, the single‐cell suspension was transferred into a differentiation medium (DIF‐medium) composed of Neurobasal, GlutaMAX, and B27 supplement containing retinoic acid (Gibco, Thermo Fisher Scientific, Grand Island, NY, U.S.A.). Cells were seeded onto glass coverslips coated with laminin (1:100 in PBS, 0.05 M) and poly‐D‐lysine (1:100 in PBS, 0.05 M) at a density of 100 cells/mm^2^ (8000 cells per coverslip) and maintained in 4‐well dishes at 37°C and 5% CO_2_. DIF‐medium was replaced every 2 days during cultivation.

### 2.4. Fixation and Immunocytochemistry

Cells grown on glass coverslips were fixed in 4% paraformaldehyde (PFA in 0.1 M NaPP) for 30 min, followed by an additional 5 min in acetone. Samples were incubated in 10% bovine serum albumin (BSA, A9418, Sigma–Aldrich, Merck, St. Louis, MO, U.S.A.) prepared in 0.1 M PBS (Sigma–Aldrich, Merck, St. Louis, MO, U.S.A.). Immunostaining was achieved through a 12 h incubation at 5°C with primary antibodies diluted in 1% BSA and 0.1 M PBS. The following antibody panel was employed: mouse monoclonal anti‐Atoh‐1 (1:1000; Ab27667—Abcam, Waltham, MA, U.S.A.), rabbit polyclonal anti‐Atoh‐1 (1:500; sc‐98520—Santa Cruz, Dallas, TX, U.S.A.), mouse monoclonal anti‐β‐tubulin (1:1000; T5293—Sigma–Aldrich, Merck, St. Louis, MO, U.S.A.), rabbit polyclonal anti‐β‐III‐tubulin (1:2000; #Ab18207—Abcam, Waltham, MA, U.S.A.), rabbit polyclonal anti‐doublecortin (DCX) (1:1000; Ab179513—Abcam, Waltham, MA, U.S.A.), mouse monoclonal anti‐GFAP (1:1000; #MAB360—Merck Millipore, Billerica, MA, U.S.A.), rabbit polyclonal anti‐MBP (1:800; #M3821—Sigma–Aldrich, Merck, St. Louis, MO, U.S.A.), rabbit polyclonal anti‐Mus‐1 (1:100; Abcam, Waltham, MA, U.S.A.), mouse monoclonal anti‐Nestin (1:800; #MAB353—Merck Millipore, Billerica, MA, U.S.A.), and rabbit polyclonal anti‐Sox‐2 (1:2000; #Ab97959—Abcam, Waltham, MA, U.S.A.).

After three rinsing steps with 0.1 M PBS, the samples were exposed for 1 h to Alexa Fluor A488‐ or A555‐conjugated secondary antibodies (1:1000; A32790, A32766, A32794, A32773; Thermo Fisher, Grand Island, NE, U.S.A.) in 1% BSA and 0.1 M PBS supplemented with DAPI (5 μg/mL; 1:5000, D9542; Sigma–Aldrich, Merck, St. Louis, MO, U.S.A.). Finally, the coverslips were rewashed, mounted on glass slides with Aqua‐Poly/Mount (18606—Polysciences, Inc., Warrington, PA, U.S.A.), and stored at 5°C in darkness until further analysis.

### 2.5. Cryosection and Immunohistochemistry

VG was fixed in 4% paraformaldehyde (PFA, Sigma–Aldrich, Merck, St. Louis, MO, U.S.A.) for 1 h and sequentially incubated in 10%, 20%, and 30% saccharose for 24 h each. The tissue was embedded in Tissue‐Tek O.C.T. compound (Sakura Finetek Europe, Umkirch, Germany), frozen in liquid nitrogen, and sectioned at 9 µm using a cryostat (CM1510S, Leica, Wetzlar, Germany). Sections were mounted on Superfrost Plus slides (Hartenstein, Wuerzburg, Germany). Postfixation was performed in 4% PFA for 5 min. Afterward slides were washed three times with TBS‐T, and blocked for 1 h in 10% BSA containing 0.3% Triton X‐100 (Sigma–Aldrich, Merck, St. Louis, MO, U.S.A.). Sections were incubated for 24 h at 4°C in 1% BSA with 0.3% Triton X‐100. Immunolabeling was performed with the following primary antibodies: rabbit polyclonal anti‐Atoh‐1 (1:500; sc‐98520—Santa Cruz, Dallas, TX, U.S.A.), mouse monoclonal anti‐β‐tubulin (1:1000; T5293—Sigma–Aldrich, Merck, St. Louis, MO, U.S.A.), rabbit monoclonal anti‐β‐tubulin (1:1000; ab179513—Abcam, Waltham, MA, U.S.A.), mouse monoclonal anti‐Nestin (1:800; MAB353—Merck Millipore, Billerica, MA, U.S.A.), and rabbit polyclonal anti‐Sox‐2 (1:2000; ab97959—Abcam, Waltham, MA, U.S.A.).

Following antibody incubation, sections were gently washed in TBS‐T and subsequently exposed for 1 h to Alexa Fluor 488‐ or 555‐conjugated secondary antibodies (1:1000; A32790, A32766, A32794, A32773—Thermo Fisher, Grand Island, NE, U.S.A.) prepared in 1% BSA/0.1 M PBS containing DAPI (5 µg/mL, 1:3000; D9542—Sigma–Aldrich, Merck, St. Louis, MO, U.S.A.). After final rinsing, the slides were coverslipped using Aqua‐Poly/Mount (18606—Polysciences, Inc., Warrington, PA, U.S.A.) and kept at 5°C in complete darkness until microscopic evaluation.

### 2.6. Statistical Analysis

All datasets were organized in Microsoft Excel 2023 V16.70 (Microsoft Corporation, Redmond, WA, U.S.A.) and analyzed statistically using GraphPad Prism 10.5 (GraphPad Software Inc., San Diego, CA, U.S.A.). Normality of data distribution was assessed with the Kolmogorov–Smirnov test. For datasets with a Gaussian distribution, group comparisons were performed using one‐way ANOVA, followed by Tukey’s multiple comparison test, and results were expressed as mean ± standard deviation (SD). When normality was not confirmed, the Kruskal–Wallis test followed by Dunn’s multiple comparison test was used, and data are reported as medians with interquartile ranges (IQRs). All tests were two‐tailed, with significance set at *p*  < 0.05. For multiple dependent comparisons, *p*‐values were adjusted using the Bonferroni correction to control for multiple testing.

## 3. Results

### 3.1. Time‐Dependent Neurosphere Formation and Viable Cell Growth Demonstrate Proliferative Potential Within VG‐Derived Cell Populations

**Figure 2:**
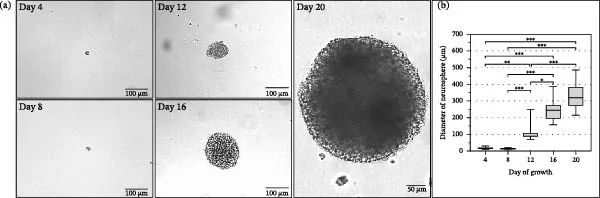
Demonstration of the proliferative capacity of VG‐derived cells by the increase in neurosphere diameter in NSC‐medium. (a) Development of primary neurospheres from VG of PND 8 rats after 4, 8, 12, 16, and 20 days in NSC‐medium. (b) Quantification of neurosphere diameter over time up to day 20 in culture. Kruskal–Wallis test followed by Dunn’s multiple comparison test was performed (*n* = 30 neurospheres per day of growth). Box plots show the median with upper and lower quartiles; whiskers indicate minimum and maximum values. Asterisks denote statistical significance:  ^∗^ *p*  < 0.05,  ^∗∗^ *p*  < 0.005,  ^∗∗∗^ *p*  < 0.001.

By day 4, the dissociated VG cells had formed spherical, free‐floating neurospheres (Figure 2a). Their diameters remained small during the early culture period, with similar medians on day 4 (15.5 µm, IQR: 14.0–18.3) and day 8 (13.5 µm, IQR: 11.0–16.5; *p*  > 0.99). A significant growth phase began after day 12, when median diameters increased to 92.5 µm (IQR: 84.6–108.0), representing a 497% increase from day 4 (*p* = 0.002). Continued growth was observed by day 16 at 244 µm (IQR: 194.0–276.0), a 1474% increase compared to day 4 and a 1707% increase from day 8 (both *p*  < 0.001). By day 20, cultures reached 318 µm (IQR: 272.0–384.0), a further 2048% increase from day 4, though the size did not differ significantly from day 16 (*p* = 0.73) (Figure 2b). These results show minimal early growth during the initial 8 days, followed by a notable and statistically significant rapid expansion of neurosphere diameters afterward.

**Figure 3:**
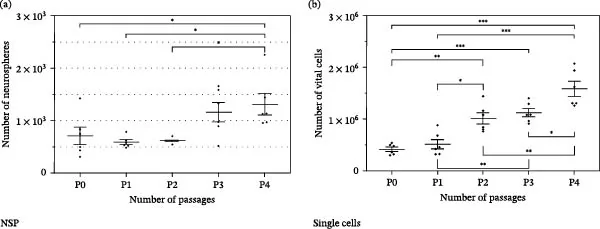
Progressive amplification of VG‐derived neurospheres and total number of viable cells across serial passages, indicating sustained proliferative potential in vitro. (a) Number of neurospheres per passage (P0–P4), showing significant increases between P0 vs. P4, P1 vs. P4, and P2 vs. P4. (b) Total number of viable cells within VG neurosphere cultures displaying a continuous and significant increase throughout passages P0–P4. One‐way ANOVA followed by Tukey’s multiple comparison test was performed. Central horizontal bars indicate the mean, and error bars represent the standard error of the mean (SEM). Each dot corresponds to one culture (*n* = 6). Asterisks indicate statistical significance:  ^∗^ *p*  < 0.05,  ^∗∗^ *p*  < 0.005,  ^∗∗∗^ *p*  < 0.001.

The persistence of neurosphere‐forming capacity during extended in vitro culture was evaluated across sequential passages (*n* = 6). At the primary stage (P0), cultures generated 706.5 ± 403.6 neurospheres per animal. Neurosphere development showed only minor fluctuations during the early passages, with a slight decrease of 17% from P0 to P1 (586.3 ± 109.9; *p* = 0.98) and small increases of 5% from P1 to P2 (617.8 ± 51.68; *p* = 0.99) and 88% from P2 to P3 (1159 ± 442.0; *p* = 0.089). Accordingly, no statistically significant differences were detected among P0, P1, P2, and P3 (P0 vs. P1: *p* = 0.98; P0 vs. P2: *p* = 0.99; P0 vs. P3: *p* = 0.20; P1 vs. P2: *p* = 0.99; P1 vs. P3: *p* = 0.06; P2 vs. P3: *p* = 0.09; Tukey’s test). A pronounced shift occurred only at the later stage, when neurosphere formation at P4 (1313 ± 494.3) exhibited an increase of 86% relative to P0, and was significantly higher compared with P0 (*p* = 0.045), P1 (*p* = 0.012), and P2 (*p* = 0.017), whereas P3 and P4 remained comparable (*p* = 0.94). These findings indicate that neurosphere‐forming cells are preserved throughout serial passaging and undergo delayed but substantial amplification, becoming statistically detectable at P4 (Figure 3a).

The number of viable cells in the culture was evaluated across serial passages (*n* = 6). The cultures contained 416,667 ± 102,307 viable cells per animal at P0. An expansion was observed during the early passages, with an increase of 23% from P0 to P1 (513,750 ± 222,136; *p* = 0.955) and a further increase of 97% from P1 to P2 (1,013,333 ± 263,717; *p* = 0.011). This growth continued from P2 to P3 (1,121,667 ± 190,543), representing an increase of 11% (*p* = 0.934), and became more pronounced at P4 (1,582,500 ± 352,516), corresponding to an overall increase of 280% compared with P0 (*p*  < 0.001). Significant differences were detected between P0 and P2 (*p* = 0.002), P0 and P3 (*p*  < 0.001), P0 and P4 (*p*  < 0.001), as well as between P1 and P2 (*p* = 0.011), P1 and P3 (*p* = 0.002), P1 and P4 (*p*  < 0.001), and P2 and P4 (*p* = 0.003), whereas P2 and P3 did not differ significantly (*p* = 0.934). These results show that viable cell numbers remain stable during the initial passages but undergo a clear and statistically significant proliferative expansion beginning at P2 (Figure 3b).

### 3.2. Expression of NSC and Progenitor Markers in VG‐Derived Neurospheres and Tissue

**Figure 4:**
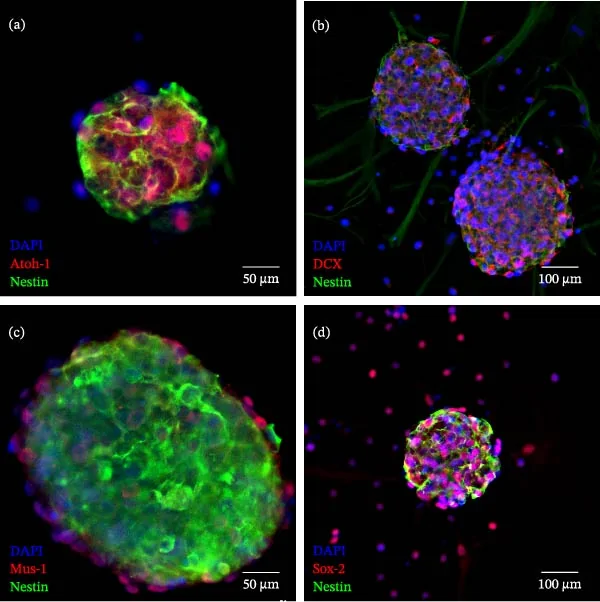
Immunocytochemical detection of NSC and neural‐lineage markers in primary VG‐derived neurospheres from PND 8 rats after 20 days in NSC‐medium. (a–d) Cells located within the neurospheres and those migrating outward from them displayed immunoreactivity for the neural progenitor marker Nestin (green), while nuclei were counterstained with DAPI (blue). (a) Cells inside the neurospheres showed nuclear localization of the transcription factor Atoh‐1 (red). (b) The neuronal migration marker DCX (red) was detected in the cytoplasm of cells within the neurospheres. (c) Mus‐1 (red), an NSC‐associated protein, was expressed in the cytoplasm of cells residing inside the neurospheres. (d) Sox‐2 (red) immunolabeling was evident in the nuclei of cells within the neurospheres.

Immunofluorescence staining demonstrated the presence of neural stem and progenitor‐associated markers in neurospheres derived from VG (Figure 4). Atoh‐1 (Figure 4a) and Musashi‐1 (Mus‐1) (Figure 4c) were detected in both the cytoplasm and in close association with the nuclei of neurosphere cells. DCX (Figure 4b) was observed in the cytoplasm, whereas Sox‐2 (Figure 4d) showed nuclear localization with colocalization with DAPI. Nestin was detected in the cytoplasm of cells within neurospheres (Figure 4a–d).

**Figure 5:**
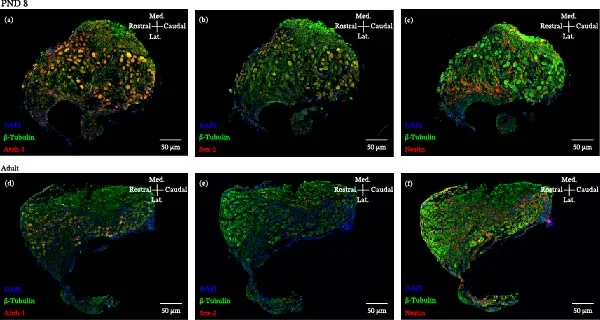
NSC and proneural marker expression in VG tissue sections from postnatal (PND 8) and adult rats. (a–f) All sections display β‐tubulin (green) in the cytoplasm and nuclear counterstaining with DAPI (blue). (a–c) Representative stainings from PND 8 rats. (d–f) Representative stainings from adult rats. (a, d) Atoh‐1 (red) showed distinct nuclear and cytoplasmic localization. (b, e) Sox‐2 (red) immunolabeling was predominantly confined to the cell nuclei. (c, f) Nestin (red) expression was detected in the cytoplasm and cellular processes of surrounding cells.

Immunofluorescence staining of VG tissue sections demonstrated the presence and spatial organization of neural stem and progenitor cell‐associated markers in vivo in both postnatal and adult animals. β‐Tubulin was detected in histological sections of the postnatal and adult VG, while DAPI labeled the nuclei (Figure 5a–f). Atoh‐1 (red, Figure 5a,d) was present in the nuclei and cytoplasm of large neuronal cell bodies. Sox‐2 (Figure 5b,e) was observed predominantly in the nuclei of smaller surrounding cells. Nestin (Figure 5c,f) was detected in cellular processes extending around these larger neuronal cell bodies.

**Figure 6:**
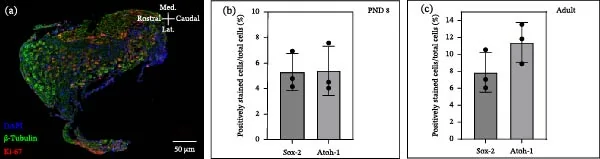
Persistence of progenitor‐associated markers in postnatal and adult VG tissue. (a) Representative immunofluorescence image of an adult vestibular ganglion section showing Ki67 (red), β‐tubulin (green), and DAPI nuclear counterstaining (blue). (b, c) Quantitative analysis of Atoh‐1‐ and Sox‐2‐positive cells in postnatal (PND 8) and adult VG tissue, respectively. Bar charts display the percentage of marker‐positive cells relative to the total number of DAPI‐positive nuclei. Values are presented as mean ± SEM (*n* = 3).

Additional immunohistochemical analyses were performed in postnatal (PND 8) and adult VG tissue. Ki67 immunoreactivity was detected qualitatively in adult VG sections (Figure 6a). The proportions of Sox‐2‐ and Atoh‐1‐positive cells relative to the total number of DAPI‐positive nuclei were quantified in postnatal and adult VG tissue (Figure 6b,c). In the PND 8 tissue, 5.29 ± 0.84% of cells were positive for Sox‐2 and 5.38 ± 1.11% for Atoh‐1. In adult tissue, 7.89 ± 1.36% and 11.42 ± 1.36% of cells stained positive for Sox‐2 and Atoh‐1, respectively (mean ± SEM; *n* = 3). The proportions of Atoh‐1 and Sox‐2 positive cells did not differ significantly between age groups.

### 3.3. Identification and Distribution of Differentiated Cell Types From VG‐Derived Neurosphere Single Cells

**Figure 7:**
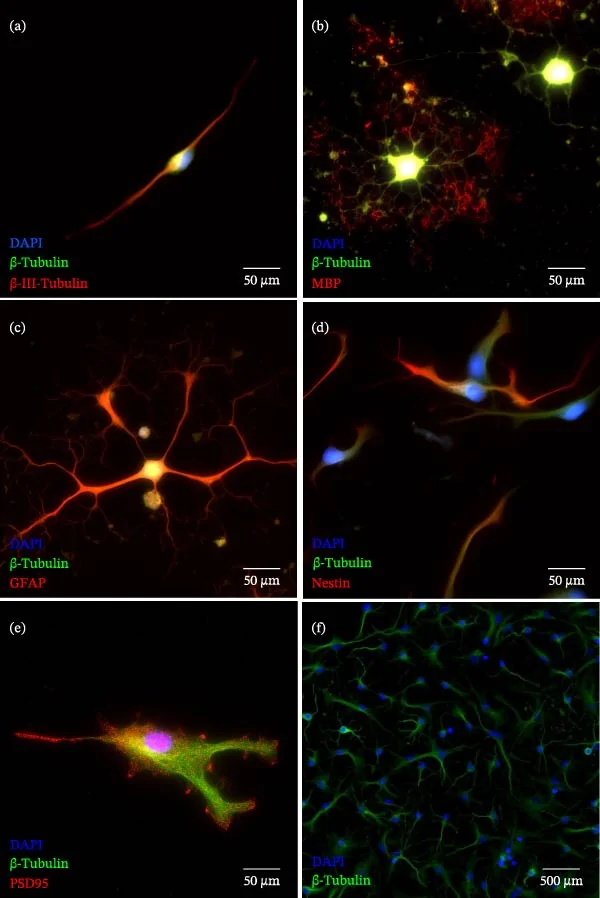
Differentiation of PND8 primary VG‐neurosphere‐derived cells into neuroectodermal cell types in DIF‐medium on glass coverslips after days 4 and 8. (a–d) All viable cells were labeled with β‐tubulin (green), and nuclei were counterstained with DAPI (blue). (a) β‐III‐Tubulin (red) marked neuronally differentiated cells. (b) MBP (red) immunoreactivity identified myelinating glial cells, showing peripheral myelination patterns. (c) Astrocytes displayed star‐shaped morphology and were detected by GFAP (red). (d) Nestin (red) labeling highlighted undifferentiated progenitor cells persisting within the cultures. (e) PSD95 (red) immunoreactivity was detected in differentiated neuronal cells. (f) Low‐magnification overview image of differentiated VG‐derived cultures showing β‐tubulin (green) and DAPI (blue).

Single cells obtained from dissociated neurospheres differentiated into various neural cell types (Figure 7). The proneuronal protein β‐III‐tubulin (Figure 7a) was detected in neuronal cell bodies and axons. Myelin basic protein (MBP), a myelination‐associated marker of myelinating glial cells (Figure 7b), was observed in cells exhibiting a myelinating glial phenotype. Glial fibrillary acidic protein (GFAP) (Figure 7c) labeled astrocytic cells, displaying characteristic star‐shaped morphologies. Nestin (Figure 7d) was detected in progenitor‐like cells originating from the dissociated single cells. In addition, immunoreactivity for the postsynaptic density protein PSD95 was detected in differentiated neuronal cells (Figure 7e), indicating the formation of synaptic structures and further supporting neuronal maturation within the differentiated cultures. A low‐magnification overview image of the differentiated cultures is provided (Figure 7f). The marker‐positive cell populations were quantified at the respective days on which expression of the corresponding lineage markers was observed in DIF‐medium, with Nestin‐ and GFAP‐positive cells detected at day 4 and βIII‐tubulin‐ and MBP‐positive cells detectable at day 8.

**Figure 8:**
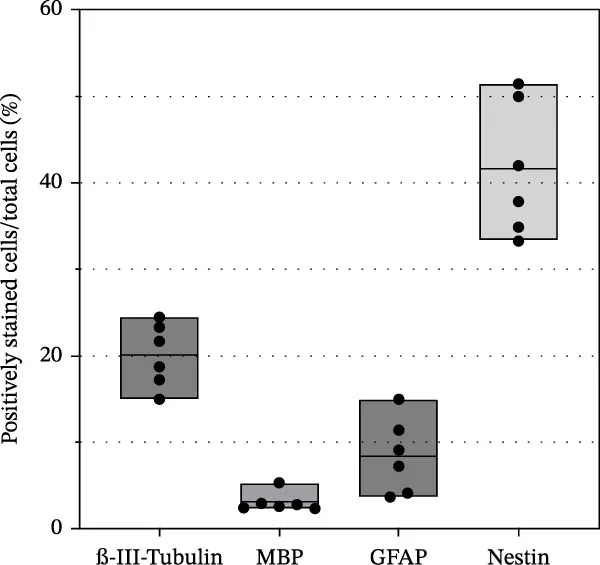
A large proportion of differentiating VG‐derived NSCs maintains a progenitor identity, while a smaller fraction adopts proneural or proglial fates. Immunocytochemical analysis was conducted on plated single VG‐derived cells from PND 8 rats in differentiation medium (DIF) on glass coverslips. The percentage of Nestin‐positive cells was highest, followed by β‐III‐tubulin–positive neurons, while only a smaller fraction of cells expressed the glial markers MBP or GFAP. Boxplots display the distribution of individual data points for each marker, with the mean indicated by horizontal bars; values are presented relative to the total number of cells per coverslip; *n* = 6.

The proportions of marker‐positive cells were quantified relative to the total number of analyzed cells. In total, 9702 cells were counted (*n* = 6). β‐III‐tubulin, labeling neuronal structures, was detected in 20.10 ± 3.68% of the cells. MBP‐positive cells exhibiting a myelinating glial phenotype were observed in 3.10 ± 1.13%. GFAP staining of astrocytic elements was detected in 8.44 ± 4.35%. Nestin, a marker of progenitor‐associated filaments, was detected in 41.58 ± 7.68% of the cells (Figure 8).

## 4. Discussion

VG‐derived cells isolated from early postnatal rats demonstrated sustained proliferation over several passages, the expression of neural progenitor markers, and multipotent differentiation into neurons, astrocytes, and myelinating glial cells in vitro. These characteristics represent the main criteria defining NSCs, namely, self‐renewal, progenitor cell generation, and neuroectodermal multilineage differentiation. As direct in situ identification of NSCs remains challenging due to the absence of a unique marker, indirect in vitro demonstration of these properties is widely regarded as the accepted approach in mammalian NSC research [36, 37]. Collectively, the findings provide robust evidence for a NSC population residing in the postnatal rat VG.

### 4.1. VG‐Derived Cells Exhibit Delayed yet Durable Proliferative Capacity Compared to Known Inner Ear NSC and Progenitor Populations

A serum‐free cell culture system with NSC‐medium was employed for the in vitro experiments. As described in Section 2.2, the medium was supplemented with the essential growth factors EGF and FGF‐2, which are known to maintain NSC viability and stimulate proliferation under nonadherent conditions [36]. Under these conditions, dissociated PND 8 VG‐derived cells reliably formed free‐floating neurospheres. Over time, the neurosphere diameter increased (Figure 2), indicating mitotic expansion within the spheres. The selection of day 14 as the passaging time point was further supported by the growth kinetics observed in Figure 2, as this interval corresponded to the phase of most pronounced neurosphere expansion and closely matched the neurosphere size range previously reported to be optimal for serial passaging [38]. Moreover, serial passaging demonstrated persistent neurosphere‐forming capacity and a steady increase in total viable cell number (Figure 3), suggesting that VG‐derived cells retain self‐renewal and proliferative capacity during passaging, a necessary feature of NSC populations [21]. Previous studies have successfully isolated sphere‐forming progenitor cells from neonatal mouse vestibular organs and cochlear spiral ganglion, enabling comparative analyses across different inner ear regions [39]. By day 12, neurospheres derived from the rat VG cells reached a median diameter of 92.5 µm (IQR 84.6–108.0) (Figure 2). This size is comparable to neurospheres derived from the mouse spiral ganglion at PND 1 after 7 days in culture (94.3 ± 30.5 µm), while neurospheres from vestibular sensory epithelia were smaller (utricle: 59.1 ± 15.2 µm; saccule: 58.0 ± 17.2 µm; and ampulla: 62.0 ± 15.1 µm) [40]. In similar spiral ganglion studies, this proliferative increase occurred earlier in culture, suggesting that VG‐derived neurospheres may require a more extended activation phase before entering a comparable growth state [41]. Differences between these studies arise in species (rat vs. mouse), developmental age (PND 8 vs. PND 1), and culture duration (12 days vs. 7 days). Recent studies have described that those factors influence neurosphere formation efficiency and growth kinetics, thereby affecting both sphere yield and proliferative output [42–44].

Previous work on neonatal murine inner ear tissues has shown that neurospheres derived from vestibular epithelia or spiral ganglion cells can be propagated for at least three serial passages, indicating sustained proliferative capacity and the presence of a stable progenitor or stem cell–like population under in vitro conditions [33, 45]. In contrast, a translational study of human inner ear tissue demonstrated a progressive reduction in neurosphere formation across passages, suggesting a more limited self‐renewal capacity and a predominance of transient progenitor cells rather than long‐term NSCs [46]. In comparison, rat VG‐derived cultures maintained neurosphere‐forming competence throughout serial passaging. They even showed an apparent increase in both neurosphere numbers and total viable cell counts from P0 to P4 (Figure 3). As discussed above, such comparisons must be interpreted with caution due to relevant interspecies differences in development, tissue composition, and responsiveness to mitogens [42]. This pattern differs markedly from the decline observed in human tissue and aligns more closely with the sustained passaging potential reported in neonatal mouse inner ear neurosphere cultures.

An essential feature of our proliferative data is the discrepancy observed between the temporal dynamics of viable cell expansion and neurosphere formation. The earlier and more pronounced rise in viable cell numbers compared to neurosphere counts (Figure 3) indicates that many proliferating cells remain as single cells or small clusters before forming detectable neurospheres. This observation is consistent with the evidence that neurospheres often arise from transit‐amplifying progenitors rather than long‐term NSCs [44]. Critical analyses of the neurosphere assay demonstrate that sphere formation frequently reflects the expansion of transit‐amplifying cells and may therefore overestimate the presence of bona fide stem cells [43]. Accordingly, the divergence between increased viable cell numbers and more moderate neurosphere formation in cell cultures (Figure 3) highlights active proliferative capacity within the VG‐derived population while emphasizing that neurosphere growth alone must be interpreted cautiously when inferring the existence of long‐term, self‐renewing NSCs [47].

Together, these findings demonstrate that VG‐derived cells retain a persistent ability to proliferate and form neurospheres across serial passages, which represents a main criterion of NSCs.

### 4.2. VG‐Derived NSC and Progenitor Marker Profiles In Vitro and In Vivo Mirror Adjacent Stem Cell Niches of the Vestibular and Auditory System

VG‐derived neurospheres expressed neural lineage–associated markers Atoh‐1, DCX, Mus‐1, Sox‐2, and Nestin when cultured in NSC‐medium (Figure 4). These markers are widely recognized indicators of the neuronal precursor identity and neurogenic potential. Atoh‐1 suggests a degree of early neuronal lineage commitment. Atoh‐1 displayed nuclear and perinuclear localization, in line with its function as a proneural transcription factor essential for sensory neuron development in the inner ear (Figure 4a) [48, 49]. DCX was detected in the cytoplasm, consistent with its well‐characterized role as a microtubule‐associated protein in immature and migrating neurons, suggesting that a subset of cells is already progressing toward early neuronal differentiation rather than remaining strictly undifferentiated (Figure 4b) [50]. Mus‐1 immunoreactivity was observed in the cytoplasm of neurosphere cells, consistent with its established role as a conserved neural precursor marker that regulates translation in undifferentiated states (Figure 4c) [51]. Sox‐2 showed predominant nuclear localization, in agreement with its role as a transcriptional regulator of NSC maintenance and self‐renewal, marking multipotent neural stem and progenitor cells (Figure 4d) [52–54]. Nestin was detected in the cytoplasm and cellular processes, consistent with its function as an intermediate filament in proliferative self‐renewing neural precursors, where it supports cytoskeletal remodeling during stem cell division (Figure 4a–d) [55, 56]. The combined expression of Sox‐2 and Nestin is a well‐established hallmark of early, undifferentiated NSC states, further supporting the immature neurogenic identity of these VG‐derived cells (Figure 4d) [57, 58].

In addition, the expression of Atoh‐1, Sox‐2, and Nestin was detected in vivo in both postnatal and adult VG tissue (Figure 5). Furthermore, Ki67 immunoreactivity was qualitatively observed in adult VG sections, and quantitative analyses confirmed the presence of Atoh‐1‐ and Sox‐2‐positive cell populations in both age groups (Figure 6). These markers allow a clear distinction between cells already entering a proneuronal differentiation pathway and those that remain in an undifferentiated, progenitor‐like state. Ki67 serves as a marker of cellular proliferation and identifies actively proliferating cells [59]. Atoh‐1 marks early neuronal lineage commitment, whereas the combined expression of Sox‐2 and Nestin is characteristic of immature NSCs and progenitor cells with self‐renewal potential [58, 60]. Atoh‐1 immunoreactivity was predominantly localized within the large neuronal somata of the VG (Figure 5a), which is consistent with the established role of Atoh‐1 in promoting differentiation and maturation of sensory neurons, as known from spiral ganglion development [48]. The spatial distribution of Sox‐2 and Nestin in the VG sections mirrors the findings in other sensory ganglia. Sox‐2‐positive cells were predominantly located in small perineuronal elements rather than within large neuronal somata (Figure 5b), consistent with reports from the dorsal root ganglion, where Sox‐2 marks satellite glial cells with latent neurogenic capacity [61]. Likewise, Nestin immunoreactivity was observed in cytoplasmic processes surrounding the neurons (Figure 5c), similar to the Nestin‐expressing progenitor‐like cells described in the dorsal root ganglion that retain postnatal neurogenic potential [62]. This alignment with other peripheral ganglia supports the interpretation that Sox‐2‐ and Nestin‐positive cells within the VG represent a nonneuronal, precursor‐competent compartment rather than vestibular neurons. While neurosphere assays and immunocytochemistry reveal that VG‐derived cells proliferate and express neurogenic markers in response to mitogen stimulation, this alone does not confirm the existence of such progenitor‐like populations in vivo. Therefore, verifying neural progenitor and stem cell markers in vivo within the native VG architecture is critical to demonstrate that these cells represent a physiologically present population rather than a culture‐induced phenotype [63]. Additionally, our in vivo data indicate a distinct spatial organization within the VG, where early neuron‐committed cells occupy the neuronal compartment, while immature and self‐renewing Sox‐2^+^/Nestin^+^ cells are localized perineuronally. Although previous studies have reported sparse Atoh‐1 and persistent Sox‐2 expression in the adult mouse vestibular sensory epithelium, comparable quantitative analyses of these progenitor‐associated cell populations in the postnatal and adult VG have not previously been reported [64, 65]. Our detection of Atoh‐1‐, Sox‐2‐, and Nestin‐positive cells in adult VG tissue (Figure 5d–f), together with the presence of Ki67 immunoreactivity (Figure 6a), indicates that progenitor‐associated cellular populations persist beyond early postnatal development. This observation further strengthens the concept that the VG may retain a latent neurogenic capacity into adulthood.

Comparable marker constellations have been described in several other neural stem and progenitor niches of the vestibular and auditory systems, providing a helpful framework for interpreting the findings of this study. In the vestibular nuclei, Sox‐2‐ and Nestin‐positive, GFAP‐coexpressing cells form a pool of quiescent and activated NSC‐like cells that give rise to new neurons under physiological conditions, establishing the vestibular nuclei as a bona fide neurogenic niche [32]. In the adult mouse inner ear, Sox‐2 expression is confined mainly to supporting cells of the organ of Corti and vestibular sensory epithelia, where it persists after ototoxic damage and is associated with a latent regenerative potential [66]. Nestin, likewise, labels stem cell–like populations in the cochlea and vestibular apparatus: in Nestin‐GFP reporter mice, it is expressed in developing spiral ganglion cells, supporting cells, Deiters’ cells, and perineural cells, indicating proliferative competence in vivo [67]. Studies in the cochlear and vestibular organs have shown that Atoh‐1 marks neurogenic lineage commitment, and its postnatal expression promotes proliferative activation in sensory pathways [68]. Mus‐1 has been implicated in maintaining progenitor states in inner ear sphere‐forming cells [40]. The persistence of Sox‐2‐positive cells in the adult VG is particularly noteworthy, as Sox‐2 remains one of the most widely used markers of adult neural stem and progenitor populations across established neurogenic niches, including the subventricular zone, dentate gyrus, and vestibular sensory epithelia [69, 70].

In summary, the detection of established NSC and progenitor markers in vitro and in vivo indicates the presence of a neurogenically competent progenitor cell population within the VG. This represents another main criterion of NSCs and parallels those described in other vestibular and auditory regions.

### 4.3. Progenitor‐Dominant Multipotent Differentiation of VG‐Derived Single Cells

Neurosphere‐derived cells were transferred into DIF‐medium (2.3) to induce differentiation, a widely used approach for neural stem and progenitor cells [71]. The neurosphere‐derived single cells differentiated into neurons, astrocytes, myelinating glial cells, and progenitor cells (Figure 7).

The cytoskeletal protein β‐III‐tubulin was used to demonstrate neuronal differentiation [72]. Among the β‐III‐tubulin–positive cells, a subset displayed a bipolar neuronal morphology with two opposing processes emerging from the soma, consistent with the well‐established bipolar organization of VG neurons in vivo. (Figure 7a) [73]. MBP immunoreactivity served to identify myelinating glial cells since MBP is a principal structural component of compact myelin in both the central and peripheral nervous systems and is associated with myelinating glial phenotypes (Figure 7b) [74]. Astrocytic differentiation was confirmed by GFAP expression as this intermediate‐filament protein is selectively enriched in mature astrocytes and is regarded as a defining cytological hallmark of the astroglial lineage (Figure 7c) [75]. Nestin‐positive cells displayed morphological and molecular features consistent with an uncommitted neural precursor phenotype, as Nestin is typically expressed during early neurodevelopment before neuronal or glial lineage specification occurs (Figure 7d) [76]. In addition, PSD95 immunoreactivity was detected in differentiated neuronal cells. PSD95 is a major scaffolding protein of the postsynaptic density and is widely used as a marker of synaptic maturation and postsynaptic specialization [77]. The detection of PSD95 therefore provides further support for neuronal maturation within the differentiated cultures.

The immunocytochemical marker panel confirmed the presence of neurons, astrocytes, myelinating glial cells, and undifferentiated precursors within the differentiated cultures (Figure 7). Notably, the predominance of Nestin‐positive cells over β‐III‐tubulin‐, GFAP‐, and MBP‐positive phenotypes indicates that a substantial portion of the population remained in a progenitor‐like state (Figure 8). In neurosphere‐derived NSC systems from the auditory pathway, such as those isolated from the medial geniculate body or the inferior colliculus, a predominantly neuronal differentiation profile has been described, with β‐III‐tubulin–positive cells forming the significant proportion of the differentiated progeny, while Nestin‐expressing precursors constitute a smaller [29, 31]. In contrast, the distribution observed in the present culture demonstrates an inverse relationship, with Nestin‐positive cells markedly exceeding the neuronal and glial compartments (Figure 8). This finding suggests that the differentiation dynamics in this NSC niche favor the persistence of progenitor characteristics over rapid lineage commitment, pointing toward niche‐intrinsic or region‐specific regulatory influences on fate acquisition. Early expression of Nestin and GFAP was detected at day 4 in DIF‐medium, whereas βIII‐tubulin and MBP became reliably detectable at day 8. Although timing can vary between niches and culture systems, this temporal pattern aligns with established observations: clonal cultures of murine cortical stem cells produce neuroblasts first and glioblasts only later under defined differentiation conditions [78–80].

Despite the use of standardized differentiation protocols and identical laboratory conditions, variation in lineage output between distinct NSC niches is anticipated, as intrinsic and niche‐imprinted properties continue to influence cellular behavior following isolation and in vitro culture. Such cultures inherently generate heterogeneous populations in which self‐renewal and lineage commitment co‐occur, resulting in variable differentiation into neuronal and glial phenotypes under identical culture conditions [81]. This phenomenon reflects that fate decisions within stem cell populations are strongly shaped by the culture environment and intrinsic single‐cell variability, making heterogeneous differentiation outcomes an expected and biologically meaningful feature of neurosphere‐derived NSC and progenitor cell systems [82]. This concept is further supported by evidence from adult NSC niches in vivo, where regional identity, dynamic state transitions, and microenvironmental cues collectively establish significant molecular and functional heterogeneity among NSCs and their progeny [83]. Moreover, the temporal dynamics of differentiation must be considered as prolonged exposure to differentiation conditions has been shown to significantly enhance neuronal commitment and functional maturation in neurosphere‐derived populations, indicating that the lineage outcome observed in this study may reflect an early or incompletely progressed differentiation stage [84].

In summary, VG‐derived cells meet the defining criterion of multipotent neuroectodermal differentiation, another main criterion of NSCs.

### 4.4. Limitations of the Study

Several limitations should be considered when interpreting these findings. Although substantial evidence for an NSC population in the VG was obtained, the cellular composition of this population remains unresolved. Inner‐ear tissues and their derived cultures contain multiple transcriptionally distinct progenitor subtypes, as shown by recent single‐cell analyses of auditory and vestibular cell populations [85]. Therefore, the in vitro approach used in this study reflects a mixture of heterogeneous progenitor states, and single‐cell RNA sequencing is a suitable strategy to dissect this heterogeneity in future studies. A further limitation concerns the uncertainty of the native activation state of VG‐derived cells. NSCs transition between quiescent and active states under physiological regulation, yet this critical aspect of niche dynamics cannot be assessed in the current mitogenic in vitro setting [86]. Intense growth‐factor stimulation likely overrides quiescence and masks regulatory mechanisms that govern activation in vivo [87]. Combining ex vivo lineage tracing or temporally controlled activation assays may therefore be required to clarify quiescent NSC behavior in the mammalian vestibular system [88]. Finally, while early postnatal tissue, like PND 8 in this study, offers high proliferative and differentiation capacity, translational relevance to mature stages remains uncertain. Age‐dependent restrictions in neurogenic potential are well documented across mammalian NSC niches [89]. Our additional in vivo analyses demonstrate the persistence of Sox‐2‐, Nestin‐, and Atoh‐1‐positive cell populations within adult VG, together with detectable Ki67 immunoreactivity. However, whether these cells retain the same proliferative, self‐renewing, and multipotent properties demonstrated in postnatal VG‐derived neurosphere cultures remains unresolved. However, vestibular function in rats develops rapidly, with measurable sensory responses by PND 6 to PND 7 and near adult‐like innervation and performance by PND 8 [90]. Our findings likely reflect a stage in which vestibular circuits are already largely established. Thus, the functional properties, activation potential, and regenerative relevance of these adult VG‐associated progenitor populations require further investigation. The present study should therefore be regarded as a foundational dataset for further investigations of adult VG plasticity and neurogenesis.

## 5. Conclusions

The present study strongly indicates that the postnatal VG contains an NSC population and provides additional evidence for the persistence within the adult VG. VG‐derived cells formed neurospheres with sustained proliferative activity, expressed neural precursor markers, and differentiated into neuronal and glial cell types in vitro. In addition, neurogenic regulators and NSC‐associated markers were detected in both postnatal and adult VG tissue, while Ki67 immunoreactivity in the adult VG further supports the presence of a persistent progenitor‐associated cellular compartment.

These findings support that VG–derived cells may provide a cellular basis for regenerative plasticity within the vestibular system. Given the growing number of identified modulators of NSC activation, proliferation, and lineage commitment, targeted manipulation of VG‐derived progenitor and stem cell populations may represent a future strategy for promoting neural repair in vestibular dysfunction [91]. To enable such therapeutic approaches, however, a more detailed understanding of the cellular states, regulatory signaling, and activation thresholds governing VG neurogenesis will be essential.

## Author Contributions

Sabine Sommerer contributed to the project coordination, cultivation and analysis of the cell cultures, microscopic analysis, statistical evaluation of the results, preparation of the immunocytological and immunohistological staining, and writing of the manuscript. Kristen Rak and Stephan Hackenberg contributed to the project supervision, coordination of the research project, and correction of the manuscript. Bjoern Spahn contributed to the supply of the cell cultures, evaluation of the differentiated single cells, and correction of the manuscript. Johannes Voelker contributed to thetechnical support for software and hardware, digital image composition, correction of the manuscript, microscopic analysis, lead of the core unit for epifluorescence microscopy, obtained the funding that supported the senior author’s dedicated research time and thus contributed substantially to the project’s realization. Jonas Engert contributed to the project coordination, project supervision, cultivation of the cell cultures, microscopic analysis, statistical evaluation of the results, and writing of the manuscript.

## Funding

This work was generously supported by the Interdisciplinary Center for Clinical Research Wuerzburg, IZKF Wuerzburg (Grants #Z‐2/CSP‐4, #Z‐2/CSP‐34, and #Z3BC/05), Beethovenstrasse 1a, Wuerzburg, D‐97080, Germany. Open Access funding enabled and organized by Projekt DEAL.

## Ethics Statement

Ethical review and approval were waived for this study because organ removal from sacrificed animals is not classified as an animal experiment under the German Animal Welfare Act (§6 Abs. 1 No. 4), although procedures complied with national guidelines for the care and use of laboratory animals.

## Conflicts of Interest

The authors declare no conflicts of interest.

## Data Availability

The data supporting the findings of this study are included in the article.
